# Hygienic

**Published:** 1894-04

**Authors:** 


### Hygienic.

FOOD HYGIENE. W. J. Moody, M.D., (Food, 1894, V., No. 1, 303).

" So we are forced to the conclusion that luxury and physical perfection are in an inverse ratio to each other." In conditions where the food supply is abundant and is obtained without manual labor, and in those where the food supply is scant and difficult to be obtained by manual labor, the results are different in the manifestations of health and vigor in the constitutions of men.

In the former, man receives more than his systemic powers can dispose of without detriment to stomach, liver, kidneys and heart; while, in the latter, the mere necessity of toil and the limited acquisition set up and maintain an order of things most favorable to digestion and assimilation. But in all cases there should be some degree of regularity in the supply of food, and some standard as to its quality; otherwise, the system may not be able to use it to subserve its ends, or, if using, the food is not adapted to the wants of the body. Among well-to-do people there is a tendency to be wiser than Nature, and a desire to improve on many of her methods. It is not necessary, say they, to take all that she offers, but only as much of the nutritious part as can be extracted and put in a concentrated form. For the nourishment of bone and muscle they take any nutriment that contains the elements of bone and muscle. So they must have sugar, fats and starch in combination and concentration, and meats and liquors, in and out of consonance with their physical states, believing that they are doing the best things for themselves that can be done. If they were truly wise they would learn the true secret of health from the stalwart workman in the open air, feeding sparingly each day on the plain fare that his hands have earned, and resting peacefully on a bed at night from which his toil has driven away all dreams and specters.

To secure among the people a large and strong physique, a robust and vigorous constitution, and a healthly and active brain, it is incumbent on parents to adopt and follow out a course of "systematic training of the young in the matter of proper feeding." If we could know the annual mortality among children in this country alone, due to overfeeding and improper feeding, "we should be appalled." If they escape death early and grow precariously to man's estate they bear the burdens picked up in the nursery or in the school, that weigh heavily on them all through life.

Aside from the intrinsic merit of the article under review, it is valuable as a warning to the many who live in luxury, and as a comfort to a greater number who earn their daily bread by incessant toil. It is a demonstration that no one can have all with immunity from human ills, and that one can have but little and yet enjoy health of mind and body. The author's idea is a true one, and can be set forth in the Latin phrase, *In media via tutissimus ibis. American Medico-Surgical Bulletin.*

